# Composites of Open-Cell Viscoelastic Foams with Blackcurrant Pomace

**DOI:** 10.3390/ma14040934

**Published:** 2021-02-16

**Authors:** Monika Auguścik-Królikowska, Joanna Ryszkowska, Maria Kurańska, Marta Wantulok, Michał Gloc, Leonard Szczepkowski, Katarzyna Dąbkowska-Susfał, Aleksander Prociak

**Affiliations:** 1 Faculty of Materials Science and Engineering, Warsaw University of Technology, Wołoska 141, 02-507 Warsaw, Poland; joanna.ryszkowska@pw.edu.pl (J.R.); marta.szewska@gmail.com (M.W.); michal.gloc.wim@pw.edu.pl (M.G.); leonardosz@interia.pl (L.S.); 2 Department of Chemistry and Technology of Polymers, Cracow University of Technology, Warszawska 24, 31-155 Cracow, Poland; maria.kuranska@pk.edu.pl (M.K.); aleksander.prociak@pk.edu.pl (A.P.); 3 Faculty of Chemical and Process Engineering, Warsaw University of Technology, Waryńskiego 1, 00-645 Warsaw, Poland; katarzyna.dabkowska@pw.edu.pl

**Keywords:** blackcurrant pomace, viscoelastic polyurethane foams, cell structure, hardness, comfort factor

## Abstract

Taking into account the circular economy guidelines and results of life cycle analyses of various materials, it was proposed to use a blackcurrant pomace filler in the production process of viscoelastic polyurethane (PUR) foams intended for application as mattresses, pillows, or elements for orthopedics. Open-cell viscoelastic PUR foams containing 10–60 per hundred polyols (php) blackcurrant pomace were prepared. It was found that after introducing the filler to the PUR foam formulation, the speed of the first stage of the foaming process significantly decreases, the maximum temperature achieved during the synthesis drops (by 30 °C for the foam containing 40 php of filler compared to unfilled foam), and the maximum pressure achieved during the synthesis of foam containing 20 php is reduced by approximately 57% compared to the foam without filler. The growth time of the foams increases with increasing the amount of introduced filler; for the foam containing 60 php, the time is extended even by about 24%. The effect of the filler on the physical, morphological, mechanical, and functional performances of PUR foam composites has been analyzed. The use of 60 php as the filler reduced the hardness of the foams by approximately 30% and increased their comfort factor from 3 to 5.

## 1. Introduction

The amount of waste resulting from various types of production is a growing problem both in Europe and worldwide [1]. According to statistical data, the global production of fresh fruit amounted to approximately 870 million tons in 2018 [2]. The dynamic increase in the production of fruit and vegetable preserves, whose average annual rate was 4.8% in the years 2011–2016, resulted in the formation of more waste from fruit and vegetable processing [3,4,5]. In the 2018/2019 season, as a result of a record harvest, the production of fruit preserves increased by 45% and reached a record level of 1.3 million tons [6]. The harvest of currants in Poland amounted to the infertile year 2017 100,000 tons, while in 2018, it was 126,000 tons [7]. Poland has become one of the world’s largest producers and exporters of semifinished products to foreign markets (fruit concentrates, frozen food, and currant preserves) [8]. The increasing amount of processed fruit and vegetables means a greater amount of postproduction waste, which is a problem for this industry sector. The management of these residues is a significant challenge, as it is associated with high disposal costs due to EU requirements in the field of environmental protection. The latest economic model promoted by the European Union is the circular economy, which assumes that production residues should be the raw material for another technology. The aim should be to minimize the amount of generated waste.

The global polyurethane (PUR) market is expected to grow by about 7.8% over the next decade and reach a value of around USD 109.26 billion by 2025 [9]. Global trends indicate that there is a need to use renewable raw materials and implement sustainable development principles in the polyurethane materials industry.

Polyurethanes are an interesting group of polymer materials that are commonly used in many applications. By choosing the type of raw materials and their proportions, it is possible to obtain a wide range of various properties of PURs that are suitable for furniture, mining, automotive, cosmetic, and many other industries. The largest PUR system is used to produce various types of foams. Viscoelastic polyurethane foams are a type of flexible polyurethane material with characteristic properties, i.e., a change in elasticity under the influence of human body heat and the ability to absorb energy and sound [10,11,12]. Currently, an increase in interest in foams with viscoelastic properties is observed. Low resilience gives these materials the ability to damp mechanical waves and absorb impact energy (up to 90%) [12]. Due to these specific properties, they are used, among others, as mattresses for home use in hospitals, hotels, as well as for orthopedic purposes [13,14].

Composite materials modified with natural fillers are a current trend in composite research. Plant fibers play a special role in modifying flexible PUR foams. There are a number of publications where various natural fillers, e.g., wood and sisal fibers, were used [15,16]. Natural fibers are characterized by a low price, they are made of renewable raw materials, and they can improve various properties of composite materials. In addition, the modified products with their participation have a lower weight compared to the same products that include, e.g., glass fibers and other ceramic fibers. In this paper, viscoelastic polyurethane foams (VEPURFs) modified with residues from the processing of blackcurrants are described. As a filler, the pomace of blackcurrant (Ribes nigrum) was applied [17,18]; such materials have not yet been tested. The use of these materials is justified by economic and ecological considerations [19].

## 2. Materials and Methods

### 2.1. Materials and Manufacturing

A series of composite materials was produced in which viscoelastic open cell polyurethane foam as described in patent PL407875-A1 was used as the matrix. The following components were used to create the materials: polyol Arcol^®^1374, reactive polyether triol with hydroxyl number LOH = 27 mg KOH/g, (Covestro AG, Leverkusen, Germany); polyol Daltocel F526, polyetherol with LOH = 128 KOH/g, (Huntsman Corporation, The Woodlands, TX, USA), polyol Rokopol^®^ M1170, block random copolymer of ethylene oxide and propylene oxide based on glycerin with LOH = 34 KOH/g, (PCC Rokita SA, Brzeg Dolny, Poland); polyol Rokopol^®^G1000, polyether polyol, glycerin-based polyoxyalkilenetriol with LOH = 158 KOH/g, (PCC Rokita SA, Brzeg Dolny, Poland); Diisocyanate Ongronate^®^4040, a mixture of monomeric isomers and oligomeric methylenediphenyl-4,4′-diisocyanate (MDI); (BorsodChem, Kazincbarcika, Hungary); foaming agents, distilled water; catalyst Jeffcat^®^ DPA, N(3-dimethylaminopropyl)-N,N-diisopropanolamine (Huntsman, Corporation, The Woodlands, TX, USA); catalyst Jeffcat^®^ ZF-10, a cycloaliphatic polyamine, (Huntsman Corporation, The Woodlands, TX, USA); surfactant Tegostab^®^B 4900, polyether-modified polysiloxane (Evonik Industries AG, Essen, Germany ).

Dried currant pomace was used as a filler, which is a by product in fruit processing in the form of pomace from AGROPOL Sp. z o.o. Fruit and Vegetable Processing Plant, Potycz, Góra Kalwaria, Poland. The delivered pomace was ground in an impact mill and then dried at 70 °C for 18 h.

PUR foams were prepared using two-component (A + B) systems. Component A, containing polyols, catalysts, surfactants, and water as well as the filler for the composites, was prepared. The component was mixed using a stirrer at 3000 rpm for 20 s. Component B was isocyanate. Using a stirrer at 3000 rpm, component A was mixed with component B for 10 s. The mixture was poured into an open rectangular mold.

Foams were produced with the isocyanate index INCO = 80. After the reaction, the materials were heated at 70 °C for one h and then seasoned for 3 days. The produced composites contained 10, 20, 30, 40, and 60% current filler.

### 2.2. Characterization Techniques

The chemical composition of the filler and composites was analyzed using absorption spectra obtained with a Fourier transform infrared (FTIR) spectrophotometer Nicolet 6700 (Thermo Electron Scientific, Waltham, MA, USA) with ATR (total reflection suppressed). Each sample was scanned 64 times in the 4000–400 cm^−1^ wavelength range. Analyses of the obtained spectra were carried out with the OMNIC 8.2.0.387 Thermo Fisher Scientific Inc. software.

The description of the chemical composition of the filler was made based on the designation according to the procedure defined by the Laboratory Analytical Procedure (LAP) in National Renewable Energy Laboratory (NREL), Determination of Structural Carbohydrates and Lignin in Biomass [20].

For the observation of the filler microstructure and composites, a scanning electron microscope (SEM) TM3000 from Hitachi Group, Tokio, Japan, was used. Samples were sputtered with a mix of palladium and gold layers. Observations were made using a 5 keV voltage.

Filler particle size analysis was carried out using the Laser Scattering Particle Size Distribution Analyzer LA-950 device, Horiba Instruments, Inc., Irvine, CA, USA. The measurements were carried out using a refractive index characteristic for cellulose (1.47) and isopropanol (1.387). The filler particle size was determined with an accuracy of ±5 µm.

The foaming process was analyzed using a FOAMAT device, Format Messtechnik GmbH, Karlsruhe, Germany, which allows characteristic parameters such as dielectric polarization, growth time, pressure, and temperature of the reaction mixture to be determined.

Thermogravimetric analysis (TGA) was performed using TGA Q500 TA Instruments, Lukens Dr, New Castle, USA. Samples of 10 ± 1 mg in the nitrogen atmosphere were tested, and they were heated at a rate of 10 °C/min from room temperature to 600 °C. The results were analyzed using the Universal Analysis 2000 version 4.7 using a TA Instrument.

Differential scanning calorimetry (DSC) was performed using DSC Q1000 TA Instruments, Lukens Dr, New Castle, USA. Measurements were made in a helium atmosphere in hermetic aluminum pans. Samples of approximately 6 mg were heated at a rate of 10 °C/min from −80 to 250 °C.

The apparent density (d) of the foams was determined in accordance with PN-EN ISO 845: 2010. The volumes of the samples were determined, the dimensions of the samples were measured with an accuracy of ±0.01 mm, and the mass was determined with an accuracy of ±0.001 g.

A mechanical compression test was carried out in accordance with the PN-EN ISO 3386−1: 2000 standard on a Zwick Z005 testing machine (Zwick Roell, Ulm, Germany). Each sample was compressed 3 times by 75% of the height before the proper measurement. The time between subsequent measurements was 300 s, giving time for foam stress relaxation and return to its original dimensions. The compressive stress values for loading and unloading were read, thus obtaining a hysteresis loop. Based on the results of the fourth compression, the foam comfort factor was determined (stress at 65% load/stress at 25% load) and stress when loading the samples at 40% deformation Compression Load Deflection CLD40, which was taken as a foam hardness parameter.

The leaching of filler particles was also evaluated due to the fact that the viscoelastic foams tested as part of the work can be washed repeatedly. The aim of the study was to verify the possibility of washing foams containing the blackcurrant pomace (BCP) filler. During the examination, the amount of solids washed out during washing was determined. Three samples of each type of foam were tested. The first samples were washed 5 times, the next were washed 10, and the last were washed 20 times. A specific amount (0.5 mL) of the same washing liquid, a similar amount of warm water, the same washing time of each sample (120 s), and the same drying position (horizontal) were used for washing. After the test, the difference in foam density was assessed.

To determine the porosity of the samples, micro computed tomography (µCT) tests were performed using the Xradia 410 CT scanner (Zeiss, Jena, Germany) with a software image reconstruction kit. This method uses X-rays and allows obtaining structural images from the inside of the material without using destructive research methods. The following scanning parameters were used in the study: a beam with an accelerating voltage of 40 kV and a power of 10 W. The exposure time for a single projection was 3 s. In total, 1261 images as a slice were taken with a 180° rotation. The scanning time was approximately 4 h for each sample. The position of the phase contrast enhancer was used. To reconstruct the received images, the built-in X-Radia reconstructor software was used. Depending on the sample, the appropriate parameters of the sample center shift and beam signal gain were introduced. The images obtained in the reconstruction were processed by the CT and a program, which used the application of the binarization threshold and the possibility of removing defects. The percentage of porosity was determined. Porosity is the percentage of closed and open pores in the total volume of the polyurethane matrix. The resolution of the method was about 20 µm.

## 3. Results and Discussion

### 3.1. The Results of the Analysis of the Structure and Chemical Structure of the Filler

**Figure 1:**
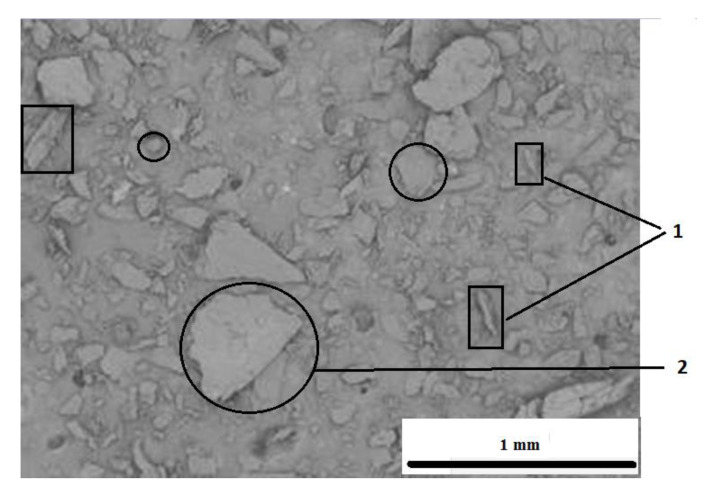
SEM image of residues from blackcurrant processing: examples of particles in the form of fibers (1), particles in the form of ovals and polygons marked with an oval (2).

Blackcurrant pomace (BCP) is the remains of fruit tissue, skin, peduncles, and seeds. After grinding, the particles of these residues take on different shapes, as shown in the SEM image (Figure 1). Parts of the peduncles take the shape of fibers, while the skin and seeds are oval and irregular polygons.

**Figure 2:**
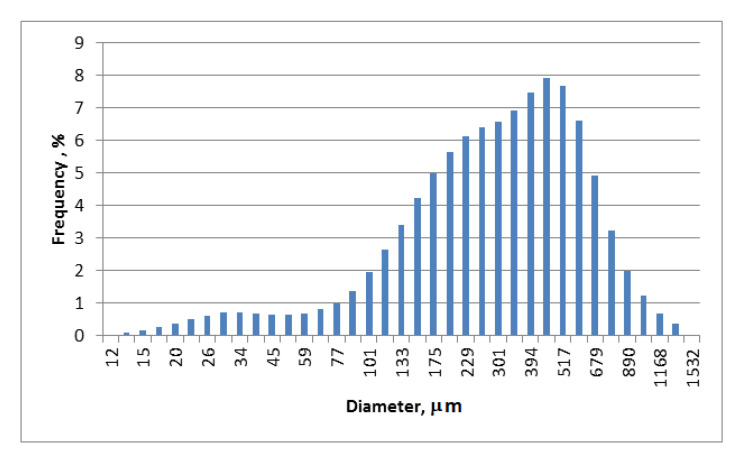
The particle size distribution of blackcurrant pomace (BCP).

Figure 2 shows the BCP particle size distribution. 

The particle size distributions were measured using a HORIBA Laser Scattering Particle Size Distribution Analyzer LA-950. BCP particles were dispersed in isopropanol and used in the funnel of the Analyzer LA-950. Sonication was performed to disperse the particles thoroughly. Particle size distribution calculations were made on the volume basis. Particle size measurements showed that the sizes of the BCP particles ranged from about 13 to 1400 μm (Figure 2). The mean size and standard deviation of the particle distributions are 337 and 221 μm, respectively. Characteristic values of particle size were accordingly about 93 μm (d10 particles of this and smaller diameter constituting 10% of the total volume of all particles in the sample), and about 663 μm (d90 particles of this and smaller diameters constituting 90% of the total volume (mass) of all particles in the sample). Probably large particles in the mass of waste are ground blackcurrant seeds.

Based on the analysis of the literature, the fillers are made of the natural substance I-row, including cellulose (30–70% by mass), hemicellulose (10–30% by mass), and lignin (2–30% by mass), and some of them also contain so-called substances II-row in an amount of 30 wt % [21]. These components constitute over 95% of the fiber weight. Blackcurrant pomace contains significant amounts of cellulose, hemicellulose, and lignin as well as pectin and fat [19,21].

The content of individual ingredients in the pomace used as a filler was evaluated by using direct methods proposed by the NREL laboratory and indirectly on the basis of TGA. Results of the determination of structural carbohydrates and lignin in biomass, the method proposed by NREL, are summarized in Table 1.

**Figure 3:**
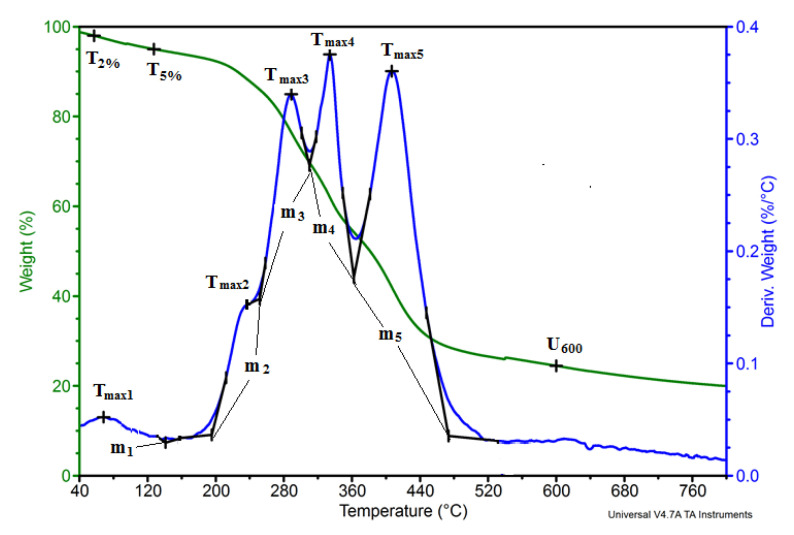
BCP thermogram.

As a result of the BCP analysis, it was found that the amount of water in the studied pomace is about 6%, and fat in the form of oils is about 27.5% by mass. Similar results were obtained for the fat content of pomace by other scientists; the values were 25.7% and 29.3 mass % according to Helgig et al. [22] and del Castillo et al. [23], respectively. According to Nawirska and Kwaśniewska [24], blackcurrant pomace contains approximately 12.0% cellulose and 25.0% hemicellulose and 59.3% lignin. In the BCPs analyzed by the NREL method, hemicellulose, cellulose, and lignin were found. The content of individual components in the pomace used as a filler was also assessed by an indirect method based on thermogravimetric analysis (Figure 3).

The process of degradation of blackcurrant pomace occurs in many stages (Figure 3). From the mass change curve, 2% and 5% loss temperatures were determined at T2% (57 °C) and T5% (127 °C), respectively. Loss of weight at 2% and 5% by weight in the case of currant pomace filler is associated with evaporation of water. The weight loss at 600 °C (U600 = 21.8%) was also determined, the value of which is related to the high carbon content in the pomace particles. Based on the data presented on the mass derivative curve, the subsequent degradation stages were determined the maximum rate of each degradation step (Vi) and the temperature at which it is reached (Tmaxi). To analyze the processes taking place in the subsequent stages of filler degradation, the beginning and end temperatures Tsi and Tei, respectively, of each stage were determined as well as the change in mass in these stages (Δmi). The results of the analyses are summarized in Table 2.

Cellulose decomposes in the temperature range 260–390 °C, and hemicellulose degrades in the range 150–280 °C. Lignin degrades in two stages: first in the range 380–440 °C, and then its aromatic components degrade around 675 °C [25,26]. The BCP degradation results were based on these degradation ranges, which are summarized in Table 2. Based on these results, it can be stated that BCP contains about 22.9% hemicellulose, 12.7% cellulose, and 35.2% lignin. The results of the BCP composition analysis based on the derivative thermogravimetry (DTG) t curve analysis are similar to the results of the Nawirska and Kwaśniewska analysis [24] when assessing hemicellulose and cellulose, but they differ significantly in assessing lignin content. The smaller amount of lignin by several percent in this filler is indicated by the results of other authors [27].

### 3.2. Foaming Behavior of PU Mixtures Containing Blackcurrant Pomace

BCP was introduced into the reference foam formulation up to 60 php (per hundred polyols), which resulted in apparent density increases with the amount of used filler (Table 3). The apparent density (D) of the foamed composites is influenced by the reaction of water introduced to the filler and the density of BCP. The apparent density of the composites does not increase significantly as is evident from the introduction of pomace to the foams with an apparent density of approximately 990 kg/m^3^ [28].

During the synthesis, the foam growth times were recorded, i.e., the time from the moment the mold is filled to the end of the foam growth process. The addition of a filler extends the growth time of the foam. After adding 60 php of filler, the growth time increased by about 25%; that is, the reaction speed leading to the creation of the foams decreased. The rate of reaction for the formation of urethane groups depends on various factors: the presence of metal compounds [29], the pH of the reaction medium [30], and the catalytic effect of the substrates and reaction products themselves [31,32]. The introduction of a natural plant filler in the form of currant pomace slowed the reactions associated with the growth of the foams, so it was decided to clarify whether this was due to a change in the pH of the reaction medium. The pH of the water mixture and 20 wt. % filler were found to be 3.5. As follows from the work of Heiss et al. [30], increasing the acidity of the reaction environment causes a clear decrease in the speed of formation of urethane bonds.

A reduced VEPUR foaming rate can also affect the current viscosity of the blend with blackcurrant pomace filler. The effect of the filler additive on the increase in composition viscosity is described in the work of Mueller et al. [33]; the authors found that with lower amounts of filler, the viscosity of the composition increases linearly with increasing amount of filler, while with higher amounts of filler, the viscosity increases non-linearly. The aspect ratio of the particles in the suspension has a strong influence on the viscosity of the composition. When comparing compositions with a similar volume content of particles, when we use spherical particles, we obtain compositions with a lower viscosity than when using particles with a higher aspect ratio. In previous works on polyurethane foams, the influences of various fillers on increasing the viscosity of the polyol premix for foam formation were demonstrated [34,35,36].

**Figure 4:**
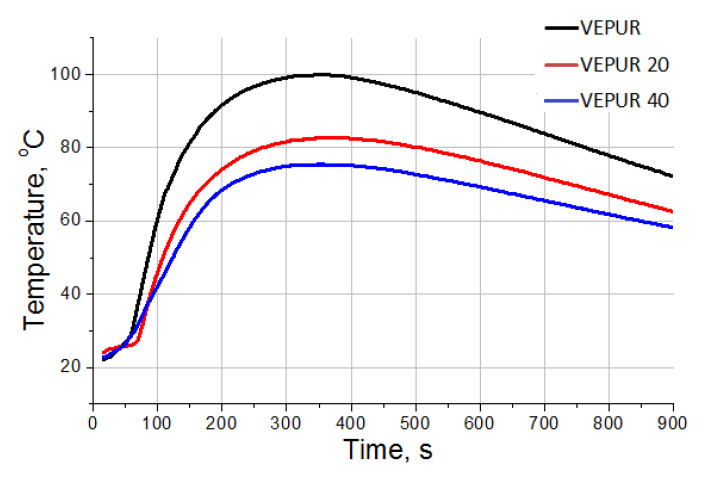
Core temperature profiles of viscoelastic polyurethane foam (VEPUR) foams and composites with 20% and 40 php BCP.

**Figure 5:**
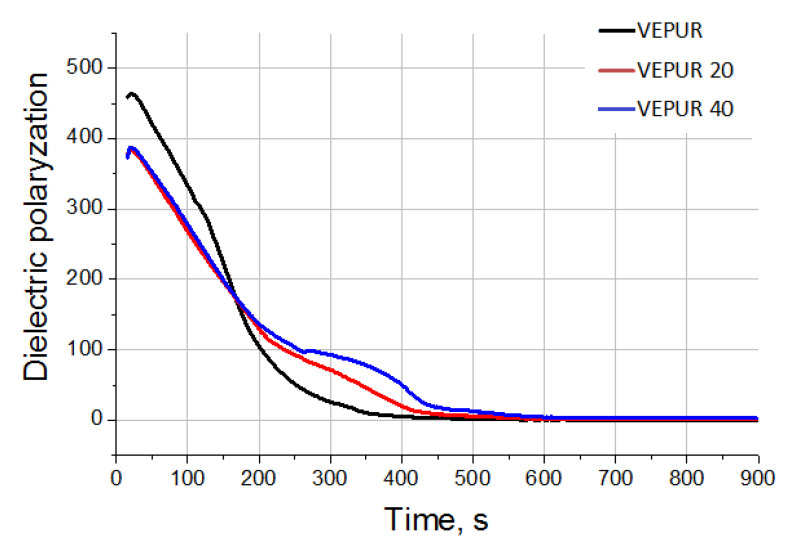
Dielectric polarization changes depending on the filler content in the polyurethane formulation.

**Figure 6:**
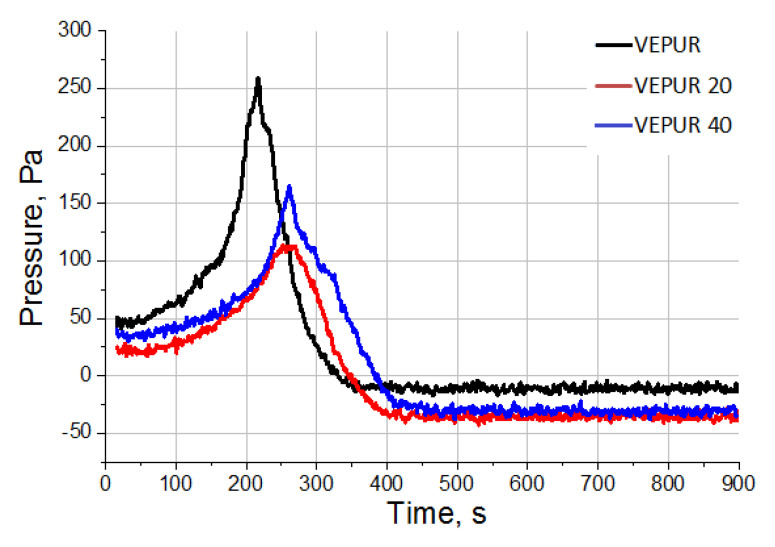
Pressure during the foaming process changes depending on the filler content in the polyurethane formulation.

In [36], it was shown that expandable graphite decreases the reactivity of the PUR systems. To confirm the observations regarding the reactivity of the tested foams, analysis of the foaming reaction of VEPUR and its composites using FOAMAT devices were carried out. Changes in temperature in the foam core (Figure 4), pressure during the reaction (Figure 5), and reaction rate (Figure 6) were analyzed. The change in temperature as a function of reaction time was analyzed because the temperature is strongly correlated with the reactivity of the PUR system. The increase of filler amount in the reaction mixture lowered the temperature rise inside the material core (Figure 4). In the work of Prociak et al. [37], it was found that powder fillers in PUR systems sometimes delay the foaming process [38], and so it is after using the current pomace filler. This is due to the fact that the pH of the reaction medium decreases and the viscosity of the substrate mixture increases after adding the filler.

The temperature in the foam core reached only approximately 80 and 70 °C for foams with, respectively, 20 and 40 php of filler, while it was about 100 °C for unfilled foam. These values are consistent with the reduced reactivity of foams after introduction of the filler and as observed during the analysis of foam growth time. For all analyzed foams, a rapid increase in temperature in the core of the foams was observed for approximately 180 s, after which the rate of temperature increase decreased, so that after approximately 250 s, it began to stabilize, and after approximately 500 s, it began to decrease. The multistage changes in the characteristics of the foam-forming reaction parameters are particularly evident in the dielectric polarization curves of VEPUR composites with BCP filler (Figure 5). Initially, up to 180 s, the dielectric polarization of composites decreased more slowly than for VEPUR, which indicated that this reaction stage in composites is slower.

After about 180 s, until the end of the synthesis process, the speed of the dielectric polarization changes for composites is higher than that for VEPUR. In foams with more filler, the reaction rate is faster than in foams with less BCP. This is probably due to the release of water bounded by the filler (added as part of the filler) and the isocyanate–water reaction, which is highly exothermic. After approximately 600 s, the dielectric polarization for all materials clearly decreases, which indicates the end of the reaction.

The introduction of BCP had an impact on the internal pressure during the foaming process (Figure 6). As observed, the addition of BCP caused a decrease in the maximum pressure during the foaming process. The filler particles may promote the release of CO_2_ formed on their surfaces during the reaction. The introduction of a 20 php filler caused a significant reduction in pressure as the foams rise. This is probably also the result of the formation of more pore nuclei around the filler particles due to the formation of CO_2_ as a result of the reaction of NCO groups with H_2_O bound to the filler particles. A larger number of germ pores promotes their opening. However, with a larger amount of filler (40 php), the pressure in the foam core is insignificantly higher than after adding 20 php. The increase in pressure in this foam is due to the higher viscosity of the reaction mixture with higher filler content. A higher viscosity of the mix promotes the formation of larger pore walls, which limits the possibility of opening the pores despite the formation of more germs. This causes an increased pressure in the foam core of VEPUR 40 compared to the VEPUR 20 foam, but it is still lower than the reference foam. The summary of the results of the analysis of the synthesis of foam and its composites with BCP are presented in Table 4.

### 3.3. Cellular Structure of PU Composite Foams

**Figure 7:**
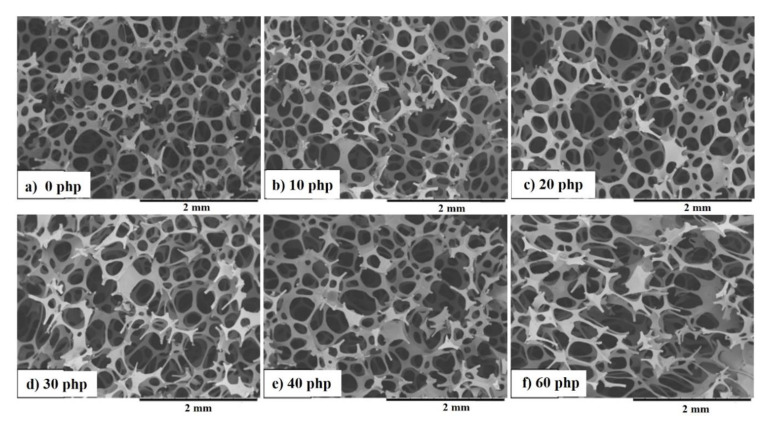
SEM images of VEPUR foam (**a**) and its composites with BCP: (**b**) 10 php, (**c**) 20 php, (**d**) 30 php, (**e**) 40 php, (**f**) 60 php.

The addition of a filler significantly reduced the temperature and pressure in the foam core, and the time until the maximum temperature and pressure was extended. The pressure change in the composites might be related to the foam’s cell opening during the foaming process leading to changes in the pore structure, as illustrated in Figure 7.

Based on SEM images, it is not possible to assess the differences in the cross-sectional structure of VEPUR foams with the addition of 10–40 php BCP. On the other hand, the structure of VEPUR 60 php foam differs significantly from the others: the pores of the foam are larger, and the pore walls are thinner than VEPUR with 10–40 php of BCP.

**Figure 8:**
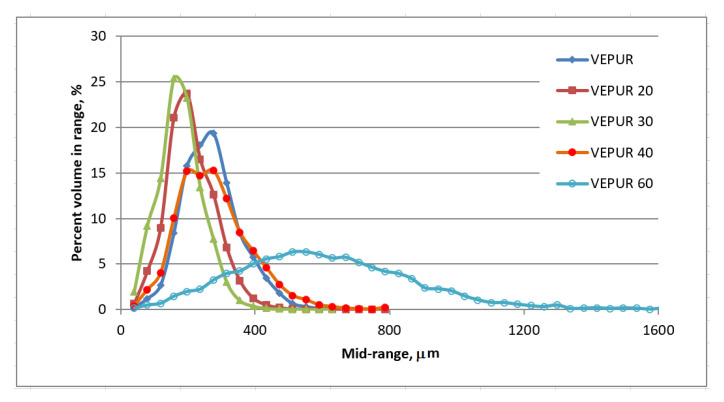
Results of pore size analysis of selected foams using tomographic analysis.

In the tested materials, the pores have a size of 0.1–1.2 mm. Pores up to 0.5 mm can be considered micropores, while pores with a size of 0.5–1.2 mm can be considered macropores. The macropores are connected by micropores with a diameter of 0.01–1 mm. All tested foams had an open-cell structure. This design guarantees adequate breathability and internal ventilation of materials. This feature is especially useful for foams used as mattresses with the possibility of washing. This facilitates and speeds up the drying process significantly. Open cell foams are sound-absorbing, flexible to light, and have a higher water permeability than closed-cell materials [8]. SEM observations of the foams did not allow for a more detailed description of the structure of the pores of the foams: therefore, analysis using computed microtomography (µCT) was performed. Selected results of this analysis are summarized in Figure 8 and Table 5.

The results of the µCT analysis confirmed that the change in pressure during foam synthesis can be associated with a change in the foam structure. The addition of a filler changed the foam pore size distribution (Figure 8). The addition of smaller amounts of 20 php, 30 php filler significantly reduced the pore size, the pore size distribution was narrower than that of unfilled foam. The reduction in pore size may be the result of the formation of more pore nuclei and the opening of cells due to the addition of filler particles [39], while the larger share of filler (40 and 60 php) caused pore size increases, and their range was wider.

With a higher filler content, the viscosity of the substrate mixture increases [40], which makes the cell opening process more difficult. At the same time, the introduction of a larger amount of filler causes the mixture to produce more small spherical bubbles. In the next step of the forming process of cells in the polymer matrix, these bubbles grow.

The features of viscoelastic foams are the result of four phenomena: relaxation, elasticity, adhesion, and pneumatic effect [39]. Two of these phenomena adhesion and pneumatic effect depend on the size of the pores, their surface development, and porosity [40].

**Figure 9:**
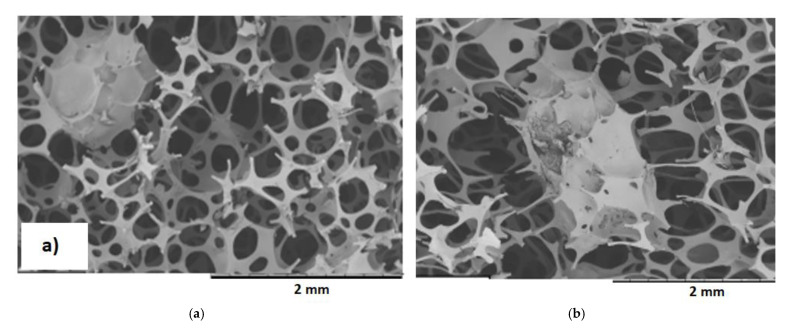
SEM images of (**a**) VEPUR 10 and (**b**) VEPUR 60.

The fine cell structure with a developed pore surface promotes the viscoelastic properties of foams. The addition of a filler with 30 php reduces the pore size, which then promotes the viscoelastic properties of VEPUR foams. The introduction of the filler causes it to be built into the pores. This phenomenon was illustrated by SEM pictures of foams with large filler particles or aggregates (Figure 9).

The foam porosity decreased after adding up to 30 php filler, and at higher levels, this quantity increases (Table 4): at 30 php, the porosity is reduced by approximately 10% compared to unfilled foam. Increasing the proportion of filler increases the porosity of modified foams (VEPUR 40 and VEPUR 60). Increasing the porosity of these foams may result from introducing water bounded in the filler structure. During the synthesis of foams at elevated temperatures, this water is released and reacts with NCO groups. During this reaction, carbon dioxide is formed, which increases the porosity of the foam. Changes in the micro- and macrostructure of foams after the introduction of the filler caused changes in their properties: therefore, the chemical structure of the foams is described.

### 3.4. Chemical Structure of PU Composite Foams

**Figure 10:**
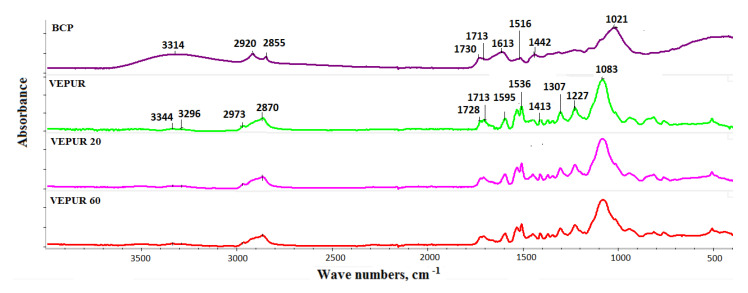
Fourier transform infrared (FTIR) spectrum of VEPUR and its composites.

FTIR spectra (Figure 10) were obtained to describe the chemical structure of VEPUR foam and its BCP foamed composites. The details of the spectra interpretation and band assignments are provided in Table 6.

**Figure 11:**
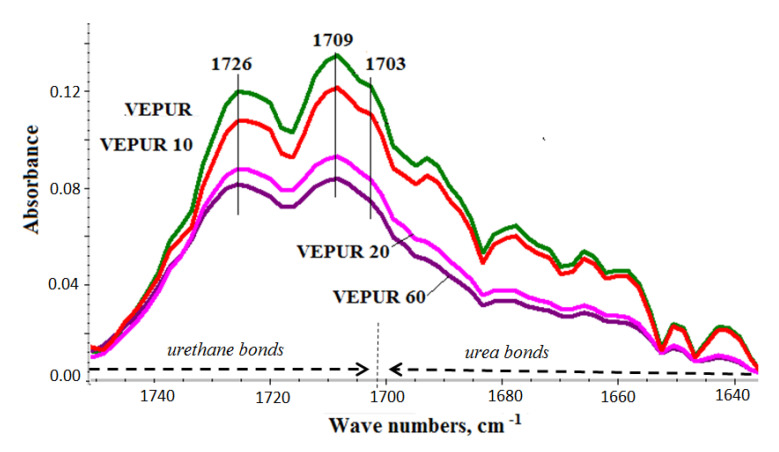
The range of the band of carbonyl groups in the analyzed materials calibrated with the vibrations of the C=C group in the aromatic ring (1600 cm^−1^).

Based on the presented spectra (Figure 10), it can be concluded that the appearance of new bands was not observed, and their positions were not shifted as a result of adding BCP. Only the intensity of the existing bands changed. The spectra of foams and their composites contain bands typical of polyurethanes (Table 6). The change in the multiplet band from the vibration of carbonyl groups in the range 1640–1750 cm^−1^ was assessed (Figure 11). Adding a different amount of filler changed the course of this band: therefore, this band was divided into component bands and the share of hydrogen bonds connecting the rigid segments, and the degree of phase separation, as well as the share of urea bonds, was calculated. The methodology for calculating these values is presented in the paper of M.M. Mazurek et al. [47]. The calculated results are presented in Table 6. It was found that the content of the hydrogen bond and the degree of phase separation decreased after adding the filler and were similar for all BCP foams despite the introduction of different amounts of filler. The introduction of the filler causes the distances between the rigid segments in composite macromolecules. Increasing the distance between the rigid segments reduces the possibility of the formation of hydrogen bonds between them. Only slight differences in the share of urethane and urea bonds were observed. With the filler, we introduce more water into the reaction medium. The greater the amount of filler, the greater the amount of water introduced to it. Water can react with isocyanate groups to produce symmetrically substituted urea and CO_2_. The rate of reaction of water with isocyanate groups is more than three times greater than that of secondary hydroxyl groups with these groups. However, the results of the FTIR analysis indicate that with the increase in the amount of filler in the tested VEPURFs, the share of urea bonds slightly decreased. The reason for the observed tendency to decrease the share of urea bonds in the tested VEPURFs may be the decreasing concentration of isocyanate groups after the first stage of the reaction (up to approximately 180 s). As can be seen from the dielectric polarization curves (Figure 5), in this stage, the VEPUR reaction is faster than for VEPUR 20 and VEPUR 40. If the reaction is faster in this stage, more NCO groups will react with the OH groups of the polyols during this time, forming urethane bonds. The differences in the level of urethane bonds formed in VEPUR 20 and VEPUR 40 in the first phase of the reaction may be influenced by the reaction temperature (Figure 4). The VEPUR 20 foam reaches a higher temperature than the VEPUR 40 foam, which increases the rate of reaction of the NCO and OH groups of polyols, favoring the formation of more urethane bonds in VEPUR 20 foam. If in the first phase of the reaction a greater number of NCO groups reacted with OH groups, there was less isocyanate left in the reaction medium, which could react with H_2_O introduced with filler I, and less urea bonds were formed in the foams (Table 7).

### 3.5. Thermal Analysis

**Figure 12:**
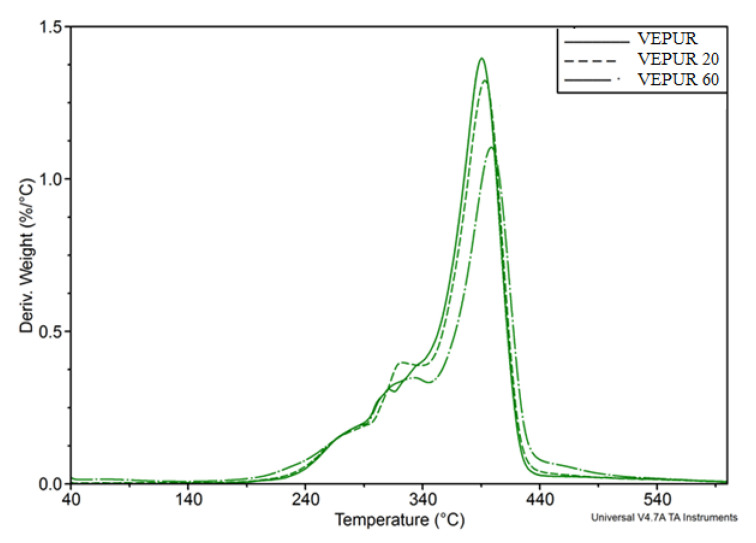
Comparison of DTG curves of VEPUR, VEPUR 20, and VEPUR 60.

Thermogravimetric analysis was also carried out for the foams, the results of derivate mass (DTG) analysis are illustrated in Figure 12. The use of a filler caused a marked change in the rate of thermal degradation of the foams.

On the basis of the TG thermogram curves, the temperature of 2% mass loss (T_2%_), temperature of 5% mass loss (T_5%_), and degradation residue at 600 °C were determined.

On the basis of the DTG curves, the temperature of the beginning and end of individual degradation stages T_i_ and T_i + 1_, as well as the weight loss at the beginning and end of each stage (m_i_ and m_i_ + 1), were determined, which allowed the determination of the change in mass during—Δm = (m_i_ + 1−m_i_). The maximum degradation rate (V_max_) and the temperature at which the maximum degradation rate (T_max_) of each stage was reached were also determined.

The course of the analysis is schematically presented in Figure 12, and the results of the analysis for all foams are presented in Table 8. This table also indicates the tendency to change for each of the foam features.

**Figure 13:**
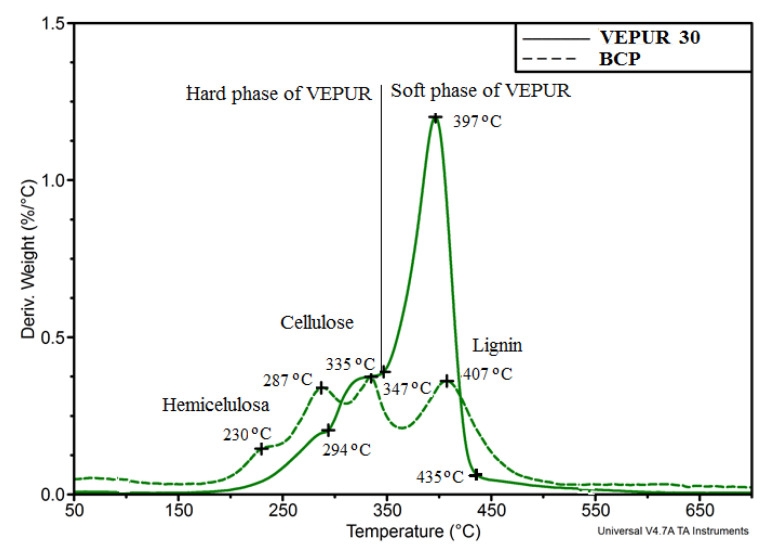
Comparison of DTG of BCP and VEPUR 30 curves.

To facilitate the interpretation of the DTG of composite thermograms in Figure 13, the DTGs of BCP and VEPUR 30 were compared.

The introduction of a filler caused a clear decrease in the 2% weight loss temperature associated with the loss of water and volatile substances contained in the foams, and the T_5%_ temperature is often considered as the onset temperature of polyurethane degradation. The more filler in the composite, the lower the T_5%_ value. The first stage of the degradation of the foam and composites occurred in the range of 227 ± 6 °C to about 295 °C, for VEPUR, VEPUR 10, and VEPUR 20 polymers, further increasing the filler content that caused the first stage to start at a lower temperature.

The second stage of degradation ends at 348 ± 3 °C, and the third ends at 432 ± 3 °C. During the first stage of the decomposition process, the hard phase of the foams degrades, probably mostly the urea bonds, and in the composites, additionally, there is a degradation of hemicellulose. The weight loss at this stage increases and changes by approximately 7.9–9.1 wt %, and the rate of degradation in the composites increases in the range 0.14−0.18%/°C. The second stage of the process is associated with the degradation of the hard phase (urethane bonds) and cellulose filler in the composites. The weight loss at this stage increases by approximately 15.7–17.5 wt %; this may result from the distribution of urethane bonds in the hard phase [48,49,50]. At this stage, the degradation rate of the composites decreases, and the weight loss changes to the range 0.41–0.35%/°C.

The third degradation stage is related to the soft phase distribution of the foams and in the lignin composites contained in the filler. In the third stage of degradation, the rate of degradation in the composites decreased by approximately 1.4–1.1%/°C with an increasing amount of filler. The weight loss in this stage decreases (65.4–53.8%). After decomposition at 600 °C, as the filler increased, the amount of ash remaining after degradation of the filler increases.

**Figure 14:**
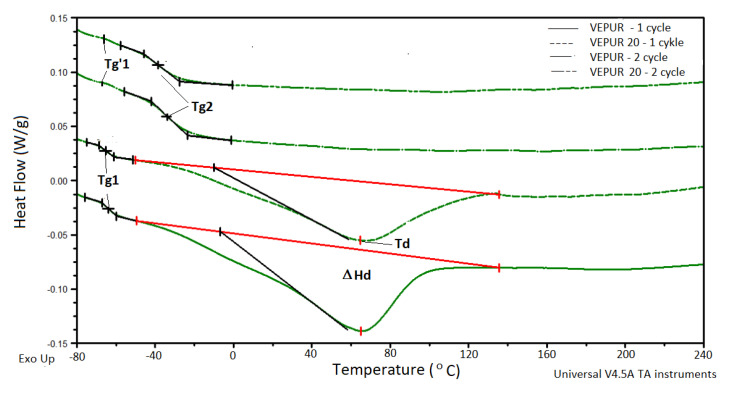
Comparison of thermograms from differential scanning calorimetry (DSC) analysis of the first and second cycle of heating.

Figure 14 presents the examples of DSC thermograms for unmodified foam and composite foams with BCP (20 php), while Table 9 summarizes the results of all sample analysis.

On the DSC thermograms of the tested materials, the occurrence of a soft phase glass transition temperature (T_g1_), a large endothermic peak associated with the change in enthalpy (ΔH_d_) with a minimum at the temperature (T_d_), was observed. This peak is associated with a change in order in the soft phase containing scattered hard domains. In the second heating cycle, two transformations associated with a soft phase glass transition (T_g’1_) and in the soft phase mixed with hard domain (T_g2_) were detected. The specific heat change (Δ c_p_) was also determined.

**Figure 15:**
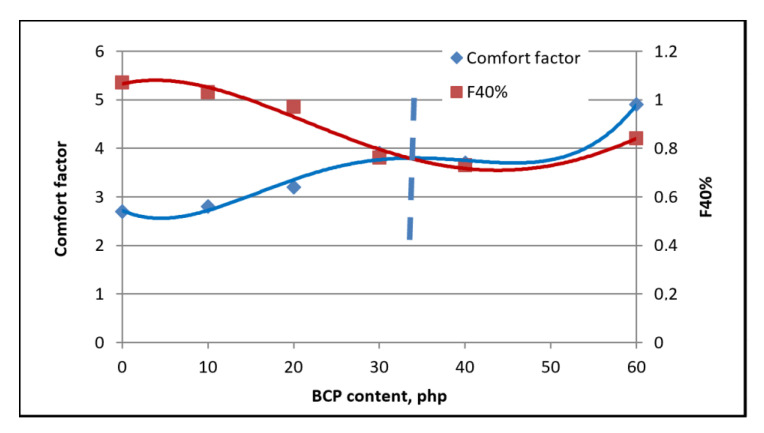
Results of analysis of strength properties determined during foam compression.

Increasing the BCP content in the foams slightly decreased the T_g1_ of their soft phase, the temperature associated with the change in order (T_d_) in the mixture of soft phase and hard domains increased, and the enthalpy of this transformation increased. The change in the specific heat change (Δc_p_) at T_g2_ indicates a lower content of the mixture of soft segments with hard domains in the composites [51,52]. Increasing the energy consumed for the phase change in T_g2_, along with increasing the amount of filler in the foams, is associated with reducing the amount of matrix in these foams. The consequence of changes in the microstructure of the foams is described with the help of FTIR, DSC, and TGA, and the macrostructure described using SEM and µCT gives changes in the strength properties of foams. Foam features were described during compression; the comfort factor and hardness of the foams were determined (Figure 15).

### 3.6. Mechanical Properties

It was observed that the comfort factor increased as the filler content increased. This is probably the result of decreased foam elasticity due to the reduction of the mobility of macromolecules in the pore walls, which is due to the incorporation of filler particles. The F40% hardness decreased for foams containing up to 30% php, after which it increased with higher filler contents. The nature of changes in foam hardness with increased filler content is the same as the nature of changes in foam porosity.

Low permanent deformation of VEPUR after compression is important, e.g., during the transport but also during the use of foam. Table 10 summarizes the results of the value of permanent deformation for all tested samples.

The use of a filler significantly increased the permanent deformation of foams by 90%. However, at 50% and 75% deformation, the permanent deformation of foam is lower than the allowable 10%.

### 3.7. Analysis of Apparent Density of Foams Washing

**Figure 16:**
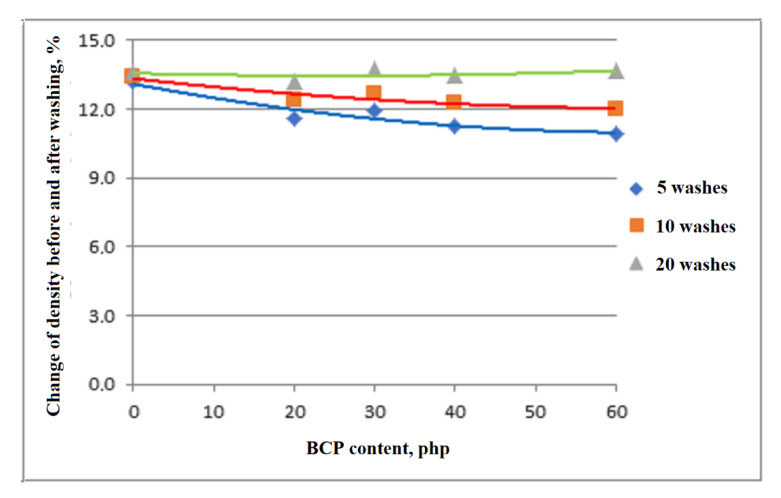
Change in foam apparent density after washing.

Viscoelastic foams can be used to make mattresses for nursing homes and people with disabilities or bed-ridden. It is preferred that these foams are amenable to repeated washing. Therefore, it was analyzed whether the tested materials could be washed. The results of the analysis of foam density changes are presented in Figure 16. After five and 10 washes, significant changes in foam density were observed, which indicates that foam particles and/or filler leached from the foams. The more the foam filler, the smaller the change in the observed material’s apparent density.

## 4. Summary and Conclusions

Blackcurrant pomace filler was used to modify the viscoelastic polyurethane foams. The filler was ground. After this process, parts of the peduncles take the shape of fibers, and the skin and seeds form oval and irregular polygons. Most filler particles are in the range from 100–900 µm. Based on a chemical analysis, it was found that the filler contains about 6% water, 28% oils, 10% cellulose, 13% hemicellulose, and 43% lignin. However, the results of the analysis using TGA indicate a similar amount of cellulose but twice the amount of hemicellulose and a much smaller amount of lignin.

Increasing the amount of filler in foams causes an elongation in the growth time of foams, the temperature and pressure in the foam core decrease during the foaming process, and the course of reaction changes. The observed changes are the result of the following: changes in the pH of the reaction medium, the filler particles acting as a germ of the pores, increasing the viscosity of the reaction mixture, the formation of additional CO_2_ resulting from the reaction of water contained in the filler, and the opening of the pores. After exceeding 30 php of filler in the foam formulation, the course of their reactions changes, but also clear changes in the foam pore size distribution are observed. Increasing the porosity of these foams may result from introducing water bounded in the filler structure. As a result of these phenomena, the foam apparent density increases and the foam porosity is reduced. This reason for the change in the nature of the phenomenon is also confirmed by an increase in pressure in the core and the image of the pore structure around the filler agglomerate.

The addition of BCP increased the apparent density of foams, but less than it would appear from using the filler with a much higher density than the density of the reference foam without BCP.

The observed increase in the comfort factor of foams can be associated with an increase in the elasticity of the material. The nature of the changes in foam hardness is associated with changes in their porosity as well as plasticization of BCP components. Permanent deformations of the analyzed foams after compression by 50% and 75% are characterized by values acceptable in industry. Moreover, the addition of BCP promotes the washability of the modified materials.

A slight increase in the rate of hard phase degradation of foams was observed, but the rate of soft phase degradation is significantly reduced, which may also be associated with the low rate of lignin degradation.

## Figures and Tables

**Table 1:** Summary of the results of the chemical analysis of the filler particles.

Component	Content, Mass %
Water and other components of the second row	6.0 ± 0.0
Oils	27.5 ± 0.5
Cellulose	10.4 ± 0.4
Hemicellulose	12.7 ± 0.3
Lignin	43.4 ± 0.5

**Table 2:** Summary of derivative thermogravimetry (DTG) curve analysis results for subsequent stages of filler particle degradation.

Stage Number/Parameter	Tsi, °C	Tei, °C	Tmaxi, °C	Vi, %/°C	Δmi, %	Description
Stage 1	40	152	68	0.05	5.9	Water
Stage 2	152	194	158	0.03	1.5	Oils or ingredients II-rows
Stage 3	194	252	234	0.15	6.6	Hemicellulose
Stage 4	252	310	288	0.34	16.3	Hemicellulose
Stage 5	310	363	334	0.38	15.7	Cellulose
Stage 6	363	474	407	0.36	25.3	Lignin
Stage 7	474	700			6.9	Decomposition of aromatic compounds
Stage 8		700			21.8	Residue after degradation

**Table 3:** Designation of tested foams, their growth time, and apparent density.

Sample Symbol	Filler Content, php	Foam Growth Time, s	D, kg/m^3^
VEPUR	0	170	45.0 ± 3.2
VEPUR 10	10	181	45.5 ± 3.7
VEPUR 20	20	199	48.9 ± 2.9
VEPUR 30	30	202	52.0 ± 3.9
VEPUR 40	40	205	55.2 ± 2.4
VEPUR 60	60	211	65.2 ± 2.6

**Table 4:** List of selected parameters of the foam synthesis process.

Parameter/Sample Symbol	VEPUR	VEPUR 20	VEPUR 40
Rise time, s	178	205	243
Max temperature, °C	100	83	76
Time occurring of max temperature, s	351	354	358
Max pressure, Pa	258	112	164
Time occurring of max pressure, s	218	264	266

**Table 5:** Summary of analysis results using micro-computed tomography (µCT).

Sample Symbol	The Range of Pore Diameter, μm	The Share of Porosity, [%]
VEPUR	263–295	90.2
VEPUR20	177–215	87.9
VEPUR30	138–177	81.0
VEPUR40	177–295	88.0
VEPUR60	492–571	91.9

**Table 6:** Assignment of functional groups in Fourier transform infrared spectroscopy by attenuated total reflectance (FTIR-ATR) spectra.

Wave Number (cm^−1^)	Possible Interpretation	Possible Interpretation
3500	ν (O–H) [36]	water or unbound polyols
3338	ν (N–H) H-bonded [41,42,43]	N–H bond
2973	ν (C–H) in CH_3_ (2970) [41,43,44,45] ν_a_ (C–H) in CH_3_ (2970) [41]	the soft segments formed from polyols
2923	ν_a_ (C–H) in CH_2_ (2925) [41,43]	
2870	ν_S_ (C–H) in CH_2_ [41,43,44,46]	
1728	ν (C=O) urethane Amide I, nonbonded [42]	urea and urethane carbonyl (C=O)
1710	ν (C=O) urethane Amide I, H-bonded [42]	
1660	ν (C=O) urea Amide I, H-nonbonded	
1600	ν (C=C) aromatic ring [41,43,44]	
1536	ν (C–N) + δ (N–H) Amide II [36]	
1510	ν (C–C) aromatic ring [41]	Urethane linkages
1455	δ (C–H) in CH_2_ [41]	
1411	ν (C=C) aromatic ring (1410) [41], ν (C–C) aromatic ring (1412–1414) [41]	
1373	ω (C–H) in CH_2_ [41,42]	
1310	δ (N–H) [41]	
1223	ν (C–O–C)	
1086	ν (C–O–C) aliphatic ether [41]	
815	δ (C–H) in aromatic ring [42]	

Note: ν stretching mode, ν_a_ asymmetric stretching, ν_S_ symmetric stretching, δ in-plane bending or scissoring, ω out-of-plane bending or wagging.

**Table 7:** Results of multiplet band analysis for vibration of carbonyl groups.

Sample Symbol/Parameter	R	DPS	Urea, %	Urethane, %
VEPUR	4.86	0.83	31	69
VEPUR10	3.56	0.78	30	70
VEPUR20	3.59	0.78	29	71
VEPUR40	3.52	0.78	27	73
VEPUR60	3.45	0.78	26	74

Where: R hydrogen bonding index; DPS degree of phase separation.

**Table 8:** Results of thermogravimetric (TG) and DTG curve analysis of foams.

Parameter/Sample Symbol	VEPUR	VEPUR 10	VEPUR 20	VEPUR 30	VEPUR 40	VEPUR 60
T_2%_, °C	248	239	237	224	212	181
T_5%_, °C	271	267	265	265	259	249
T_1_, °C	227	227	227	223	209	203
T_max1_, °C	276	264	281	281	278	281
V_max1_, %/°C	0.17	0.16	0.14	0.18	0.17	0.18
T_2_, °C	295	299	299	296	295	295
Δm_1_, %	8.0	7.8	7.9	8.2	8.0	9.1
T_max2_, °C	309	320	324	322	331	333
V_max2_, %/°C	0.30	0.40	0.41	0.36	0.37	0.35
T_3_, °C	348	346	346	347	350	351
Δm_2_, %	15.7	16.7	16.7	16.8	17.0	17.5
Δm_1_ + Δm_2_	23.7	24.5	24.6	25.0	25.0	26.6
T_max3_, °C	391	393	395	396	399	398
V_max3_, %/°C	1.40	1.32	1.28	1.20	1.20	1.10
T_4_, °C	426	428	428	431	435	432
Δm_3_, %	65.4	62.9	62.3	59.7	57.7	53.8
U_600_, %	6.7	7.6	8.3	9.7	10.7	12.3

**Table 9:** Results of the DSC analysis of the examined materials.

Sample Symbol/Parameter	T_g1_ [°C]	T_d_ [°C]	ΔH_d_ [J/g]	T_g’1_ [°C]	T_g2_ [°C]	Δ c_p_ [J/g°C]
VEPUR	−63.8	65.1	64.7	−60.9	−33.7	0.318
VEPUR 10	−65.5	65.8	64.0	−61.5	−38.7	0.243
VEPUR 20	−65.4	70.3	52.3	−61.7	−38.5	0.239
VEPUR 30	−65.4	71.2	61.7	−61.4	−38.1	0.229
VEPUR 40	−65.8	75.4	67.3	−61.8	−38.2	0.208
VEPUR 60	−66.2	78.8	77.1	−62.2	−38.1	0.192

**Table 10:** Results of measurements of permanent deformation after compression of produced foams.

Sample Symbol/Property	Deformation		
	50%	75%	90%
	Compression Set, %		
VEPUR	2.8	1.1	1.4
VEPUR 10	1.8	1.7	44.8
VEPUR 20	2.4	2.8	82.2
VEPUR 30	1.9	2.6	77.9
VEPUR 40	3.5	5.4	82.7
VEPUR 60	2.2	4.3	74.2

## Data Availability

Data sharing is not applicable to this article.

## References

[1] Ostrowska M., Winners C.O.S. Use of Waste for Food Production. https://bridge.gov.pl/aktualnosc/pokaz/wykorzystanieodpadow-do-produkcji-zywnosci.

[2] Global Production of Fresh Fruit From 1990 to 2019. https://www.statista.com/statistics/262266/global-production-of-fresh-fruit.

[3] Resulting Estimate of the Main Agricultural and Horticultural Crops in 2019. https://stat.gov.pl/obszary-tematyczne/rolnictwo-lesnictwo/uprawy-rolne-i-ogrodnicze/wynikowy-szacunek-glownych-ziemioplodow-rolnych-i-ogrodniczych-w-2019-roku,5,18.html.

[4] Data of Polish Fruit and Vegetable Production. http://ksow.gov.pl/rynki-rolne/news/entry/8426-ostateczne-dane-polskiej-produkcji-owocow-i-war.html.

[5] Predicting a World Record for the Production of Berries. http://ksow.gov.pl/rynki-rolne/news/entry/8494-nadal-przewidywania-rekordu-swiatowej-produkcji.html.

[6] IERiGŻ Report: Fruit and Vegetable Market and Fruit and Vegetable Preserves. https://www.portalspozywczy.pl/owoce-warzywa/wiadomosci/raport-ierigz-rynek-owocow-i-warzyw-oraz-przetworow-owocowo-warzywnych,173692.html.

[7] KOWR: Poland is the largest producer of currants in the European Union. https://www.sadyogrody.pl/owoce/101/kowr_polska_najwiekszym_producentem_porzeczek_w_unii_europejskiej,18662.html.

[8] Polish Power in the Production of Black Currants in the World-Dr hab. Stanisław Pluta. https://apetytnapolskie.com/polska-potega-w-produkcji-porzeczek-czarnych-w-swiecie-dr-hab-stanislaw-pluta-prof-io/#_ftn1.

[9] Global Polyurethane Market Analysis & Trends-Industry Forecast to 2025. http://www.prnewswire.com/news-releases/global-polyurethane-market-analysis--trends---industry-forecast-to-2025-300431333.html.

[10] Prociak A., Rokicki G., Ryszkowska J. (2014). Materiały poliuretanowe. Wydawnictwo Naukowe.

[11] Vaughan B.R., Wilkes G.L., Dounis D.V., McLaughlin C. (2011). Effect of vegetable-based polyols in unimodal glass-transition polyurethane slabstock viscoelastic foams and some guidance for the control of their structure–property behavior. Int. J. Appl. Polym. Sci..

[12] Petrovic Z. (2008). Polyurethanes from Vegetable Oils. Polym. Rev..

[13] Kuranska M., Prociak A. (2012). Porous polyurethane composites with natural fibres. Compos. Sci. Technol..

[14] Pelletier H., Belgacem N., Gandini A. (2006). Acrylated vegetable oils as photocrosslinkable materials. J. Appl. Polym. Sci..

[15] Soykeabkaew N., Supaphol P., Rujiravanit R. (2004). Preparation and characterization of jute- and flax-reinforced starch-based composite foams. Carbohydr. Polym..

[16] Zmarlicki K., Brzozowski P. Conditions for the Production of Gooseberries, Blackcurrants and Highbush Blueberries. http://www.inhort.pl/files/program_wieloletni/PW_2015_2020_IO/spr_2016/5.1_2016_Raport_owoce.pdf.

[17] Zawirska A. (2007). Zagospodarowanie odpadów z przemysłu owocowo-warzywnego. Przemysł Fermenstacyjny i Owocowo-Warzywny.

[18] Król S., Nawirska A. (2003). Co-removal of metal ions with pomace in a dynamic system. Technol. Aliment..

[19] Sluiter A., Hames B., Ruiz R., Scarlata C., Sluiter J., Templeton D., Crocker D. Determination of Structural Carbohydrates and Lignin in Biomass. https://www.nrel.gov/docs/gen/fy13/42618.pdf.

[20] Layth M., Ansari M.N.M., Pua G., Mohammad J., Islam S.M. (2015). A Review on Natural Fiber Reinforced Polymer Composite and Its Applications. Int. J. Polym. Sci..

[21] Dvaranauskaite A., Venskutonis P.R., Raynaud C., Talou T., Vi’kelis P., Sasnauskas A. (2009). Variation in essential oil composition in buds of six black currant (*Ribes Nigrum L*.) cultivars at various development phases. Food Chem..

[22] Helbig D., Bohm V., Wagner A., Schubert R., Jahreis G. (2008). Berry seed press residues and their valuable ingredients with special regard to black currant seed press residues. Food Chem..

[23] Del Castillo R.M.L., Dobson G., Brennan R., Gordon S. (2002). Genotypic variation in fatty acid content of blackcurrant seeds. J. Agric. Food Chem..

[24] Nawirska A., Kwaśniewska M. (2005). Dietary fibre fractions from fruit and vegetable processing waste. Food Chem.

[25] Shen D., Xiao R., Gu S., Zhang H., van de Ven T., Kadla J. (2013). The Overview of Thermal Decomposition of Cellulose in Lignocellulosic Biomass. Cellulose-Biomass Conversion.

[26] Fengel D. (1969). The ultrastructure of cellulose from wood. Wood Sci. Technol..

[27] Anioła J., Górecka D., Gawęcki J. (2005). Composition and selected physicochemical properties of micronizated high-fiber preparations. Żyw. Człow. Metab.

[28] Malińska K. (2012). Laboratory determination of air-filled porosity for composting materials. Inż. Ochr. Środ..

[29] Saunders J.H., Frisch K.C. (1968). Polyurethanes.

[30] Heiss H.L., Combs F.P., Gemeinhardt P.G., Saunders J.H., Hardy E.E. (1959). Influence of Acids and Bases on Preparation of Urethane Polymers. Ing. Eng Chem..

[31] Olczyk W. (1968). Poliuretany.

[32] Lipatowa T.E. (1974). Kataliticzeskaja Polimeryzacja Oligomerow i Formirowanie Polimernych Setok.

[33] Mueller S., Llewellin E.W., Mader H.M. (2010). The rheology of suspensions of solid particles. Proc. R. Soc. A Math. Phys. Eng. Sci..

[34] Kurańska M., Barczewski M., Uram K., Lewandowski K., Prociak A., Michałowski S. (2019). Basalt waste management in the production of highly effective porous polyurethane composites for thermal insulating applications. Polym. Test..

[35] Kurańska M., Prociak A., Cabulis U., Kirpiuks M. (2015). Water-blown polyurethane polyisocyanurate foams based on bio-polyols with wood fibers. Polimery.

[36] Kurańska M., Prociak A., Cabulis U., Kirpluks M., Ryszkowska J., Auguścik M. (2017). Innovative porous polyurethane-polyisocyanurate foams based on rapeseed oil and modified with expandable graphite. Ind. Crops Prod..

[37] Prociak A., Malewska E., Bąk S. (2016). Influence of Isocyanate Index on Selected Properties of Flexible Polyurethane Foams Modified with Various Bio-Components. J. Renew. Mater..

[38] Landers R., Knott W., Boinowitz T. New Cell Opening Strategies for TDI 80 Viscoelastic Foams by Additive Means. Proceedings of the Conference Polyurethanes.

[39] The Adjustment of Physical Properties of Viscoelastic Foam–the Role of Different Raw Materials. https://www.pu-additives.com/product/pu-additives/downloads/adjustments-of-physical-properties.pdf.

[40] The Importance of Cell Structure for Viscoelastic Foams. https://www.pu-additives.com/product/pu-additives/downloads/importance-of-cell-structure-for-viscoelastic-foams.pdf.

[41] Kurańska M., Prociak A., Michałowski S., Cabulis U., Kirpluks M. (2016). Microcellulose as a natural filler in polyurethane foams based on the biopolyol from rapeseed oil. Polimery.

[42] Soto G.D., Marcovich N.E., Mosiewicki M.A. (2016). Flexible polyurethane foams modified with biobased polyols: Synthesis and physical-chemical characterization. J. Appl. Polym. Sci..

[43] Dillon J.G. (1989). Infrared Spectroscopic Atlas of Polyurethanes.

[44] Tu Y.C., Suppes G.J., Hsieh F.H. (2009). Thermal and mechanical behavior of flexible polyurethane-molded plastic films and water-blown foams with epoxidized soybean oil. J. Appl. Polym. Sci..

[45] Hatchett D., Kinyanju J.M., Sapochak L. (2007). FTIR Analysis of Chemical Gradients in Thermally Processed Molded Polyurethane Foam. J. Cell. Plast..

[46] Zieleniewska M., Szczepkowski L., Krzyżowska M., Leszczyński M.K., Ryszkowska J. (2016). Rigid polyurethane foam composites with vegetable filler for application in the cosmetics industry. Polimery.

[47] Mazurek M.M., Tomczyk K., Auguścik M., Ryszkowska J., Rokicki G. (2015). Influence of the soft segment length on the properties of water-cured poly(carbonateurethane-urea)s. Polym. Adv. Technol..

[48] Bernardini J., Angullesi I., Coltelli M.B., Cinelli P., Lazzeri A. (2015). Optimizing the lignin based synthesis of flexible polyurethane foams employing reactive liquefying agents. Polym. Int..

[49] Demirci F., Yildirim K., Kocer H.B. (2018). Antimicrobial open-cell polyurethane foams with quaternary ammonium salts. J. Appl. Polym. Sci..

[50] Zhang L., Jeon H.K., Malsam J., Herrington R., Macosko C.W. (2007). Substituting soybean oil-based polyol into polyurethane flexible foams. Polymery.

[51] Sonnenschein M.F., Prange R., Schrock A.K. (2007). Mechanism for compression set of TDI polyurethane foams. Polymer.

[52] Cassel R.B., Wadud B.S. (2005). Thermal and Mechanical Analysis of Polyurethane Memory Foam. Am. Lab..

